# LKB1 promotes cell survival by modulating TIF-IA-mediated pre-ribosomal RNA synthesis under uridine downregulated conditions

**DOI:** 10.18632/oncotarget.6224

**Published:** 2015-10-25

**Authors:** Fakeng Liu, Rui Jin, Xiuju Liu, Henry Huang, Scott C. Wilkinson, Diansheng Zhong, Fadlo R. Khuri, Haian Fu, Adam Marcus, Yulong He, Wei Zhou

**Affiliations:** ^1^ Department of Hematology and Medical Oncology, Emory University School of Medicine, Atlanta, GA, USA; ^2^ Department of Gastrointestinal Surgery, The First Affiliated Hospital of Sun Yat-sen University, Guangzhou, P.R. China; ^3^ Graduate Program in Cancer Biology, Emory University, Atlanta, GA, USA; ^4^ Department of Medical Oncology, Tianjin Medical University General Hospital, Tianjin, P.R.China; ^5^ Department of Pharmacology, Emory University School of Medicine, Atlanta, GA, USA; ^6^ Department of Pathology and Laboratory Medicine and Department of Human Genetics Emory University School of Medicine, Atlanta, GA, USA

**Keywords:** pre-ribosomal RNA synthesis, TIF-IA, serine/threonine kinase 11, targeted therapy, tumor suppressor

## Abstract

We analyzed the mechanism underlying 5-aminoimidazole-4-carboxamide riboside (AICAR) mediated apoptosis in LKB1-null non-small cell lung cancer (NSCLC) cells. Metabolic profile analysis revealed depletion of the intracellular pyrimidine pool after AICAR treatment, but uridine was the only nucleotide precursor capable of rescuing this apoptosis, suggesting the involvement of RNA metabolism. Because half of RNA transcription in cancer is for pre-ribosomal RNA (rRNA) synthesis, which is suppressed by over 90% after AICAR treatment, we evaluated the role of TIF-IA-mediated rRNA synthesis. While the depletion of TIF-IA by RNAi alone promoted apoptosis in LKB1-null cells, the overexpression of a wild-type or a S636A TIF-IA mutant, but not a S636D mutant, attenuated AICAR-induced apoptosis. In LKB1-null H157 cells, pre-rRNA synthesis was not suppressed by AICAR when wild-type LKB1 was present, and cellular fractionation analysis indicated that TIF-IA quickly accumulated in the nucleus in the presence of a wild-type LKB1 but not a kinase-dead mutant. Furthermore, ectopic expression of LKB1 was capable of attenuating AICAR-induced death in AMPK-null cells. Because LKB1 promotes cell survival by modulating TIF-IA-mediated pre-rRNA synthesis, this discovery suggested that targeted depletion of uridine related metabolites may be exploited in the clinic to eliminate LKB1-null cancer cells.

## INTRODUCTION

The nucleolus is the nuclear subdomain in which RNA polymerase I (RNA Pol I) transcription and the assembly of ribosomal subunits occur in eukaryotic cells. An abnormal nucleolus has been used as a marker for aggressive cancer for over 100 years [1], and more recent studies have linked dysregulated nucleolar morphology with the hyperactivation of ribosomal RNA (rRNA) transcription [2]. It was estimated that approximately 80% of cancer cell energy consumption is used for rRNA biogenesis [3], and the synthesis of pre-rRNA, the first event in this process, is efficiently regulated mostly through the reversible modification of RNA Pol I transcription factors [2]. 30-50% of RNA transcription in cancer cells is for pre-rRNA synthesis [4], and accelerated rRNA synthesis is one of the most important molecular alterations in cancer cells [5].

RNA polymerase I-specific transcription initiation factor (TIF-IA, or RRN3) mediates the interaction between RNA Pol I and the transcription factor SL1/UBF (Upstream Binding Factor) to form the pre-initiation complex at the ribosomal DNA promoter, which is essential for the initiation of pre-rRNA transcription [2]. Various oncogenic alterations have been associated with increased TIF-IA-mediated pre-rRNA synthesis. Activated AKT was found to enhance TIF-IA-mediated pre-rRNA synthesis by stabilizing TIF-IA and altering TIF-IA phosphorylation at Ser170 and Ser172 through casein kinase IIα (CK2α) in leukemia cells [6]. Growth factors induce pre-rRNA synthesis by activating MAPK signaling that targets through TIF-IA, and the phosphorylation of TIF-IA at Ser649 by ribosomal s6 kinase (RSK) and Ser633 by extracellular-signal-regulated kinase (ERK) on the C-terminus promotes RNA Pol I transcription initiation [7]. A study with rapamycin also suggests that mammalian target of rapamycin (mTOR) might activate TIF-IA by increasing phosphorylation at Ser44 and reducing phosphorylation at Ser199, which enhances the formation of the Pol I pre-initiation complex at the rDNA promoter [8]. Interestingly, the depletion of TIF-IA not only abrogated RNA Pol I transcription but also promoted disintegration of the nucleolus, cell cycle arrest and apoptosis, suggesting that TIF-IA may be a potential target for cancer therapy [9].

5-aminoimidazole-4-carboxamide riboside (AICAR) was reported to induce caspase-3 cleavage in LKB1-null mouse embryonic fibroblast (MEF) cells and in cervical cancer cells [10, 11], although the underlying mechanism(s) were not explored. Inside a cell, AICAR is converted to AICAr monophosphate (ZMP) by adenosine kinase, and ZMP can act as an AMP analog to activate AMP-activated protein kinase (AMPK) in an LKB1-dependent manner. Even though AICAR-induced activation of AMPK has been shown to promote apoptosis in several cell lines, this type of apoptosis is usually dependent on the activation of AMPK [12, 13], which is unlikely to be the mechanism in LKB1-null cells [14, 15]. In this study, we developed model systems in NSCLC cell lines by either depleting the expression of LKB1 by RNAi or restoring the expression of LKB1 in LKB1-null cells. These cell lines are of diverse genetic background, and they allowed us to identify a novel role of LKB1 kinase activity in regulating TIF-IA-mediated rRNA synthesis after AICAR treatment.

## RESULTS

### AICAR treatment induces apoptosis specifically in LKB1 kinase-deficient NSCLC cells

**Figure 1:**
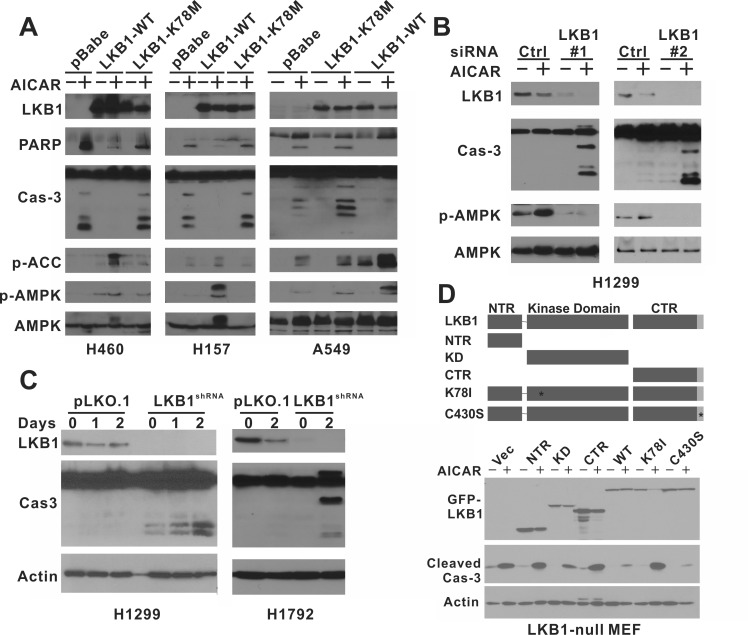
AICAR treatment induces apoptosis specifically in LKB1-null NSCLC cells **A.** H460 isogenic cell lines were treated with 1 mM AICAR for 24 hrs. H157 and A549 isogenic cells were treated with 2 mM AICAR for 24 hrs and 48 hrs, respectively. K78M: kinase-dead mutant. **B.** H1299 cells were treated with LKB1 siRNA or control siRNA. 2 mM AICAR was added 48 hrs after siRNA transfection. **C.** H1299 and H1792 cells were treated with control lentivirus or virus containing LKB1-shRNA to obtain stable cell lines. Cells were treated with 2 mM AICAR for 48 hrs. **D.** LKB1-null MEF cells were infected with retrovirus containing indicated LKB1 constructs to obtain stable cell lines. NTR: N-terminal Region; KD: Kinase Domain; CTR: C-terminal Region; K78I: Kinase-Dead mutant; C430S: farnesylation motif mutation to inhibit farnesylation. Cells were treated with 1 mM AICAR for 24 hrs. In all experiments, cell lysates were collected after the indicated time of AICAR treatment for immunoblot analysis using indicated antibodies. Antibody against total caspase-3 was used in panel a, b, and c, and antibody against cleaved caspase-3 was used in panel d.

To determine whether AICAR induced apoptosis specifically in LKB1-deficient NSCLC cells, we restored LKB1 expression in LKB1-null H460, H157 and A549 cells using a retrovirus expressing wild-type LKB1 protein. These cell lines were also infected with either an empty pBabe virus or a virus that expressed an LKB1-K78M kinase-dead mutant. The phosphorylation of AMPK at Thr172 and acetyl-coA carboxylase (ACC) at Ser79 were used as surrogate markers of LKB1 kinase activity under AICAR treatment conditions (Figure 1). AICAR-induced poly ADP ribose polymerase (PARP) and caspase-3 cleavage were only observed in cell lines treated with the control virus but not in cells that expressed wild-type LKB1 (Figure 1A). Furthermore, the depletion of LKB1 by two different siRNA in LKB1-wild type H1299 cells also rendered them sensitive to AICAR-induced caspase-3 cleavage (Figure 1B). Similarly, stable depletion of LKB1 by lentiviral-shRNA promoted AICAR-induced caspase-3 cleavage in LKB1-wild type H1299 and H1792 cells (Figure 1C). The appearance of apoptotic H1299 cells after RNAi treatment was also confirmed by Annexin-V/7AAD flow cytometry analysis (Supplemental Figure 1). To comprehensively evaluate the various functional regions of LKB1 in this process, we also expressed LKB1-missense or truncation mutants in LKB1-null MEF cells with a GFP-tag. The expression of wild-type or C430S farnesylation mutant LKB1, but not the K78I kinase-dead mutant, was sufficient to attenuate AICAR-induced caspase-3 cleavage (Figure 1D). Similarly, the expression of a different K78M kinase-dead mutant also failed to attenuate caspase-3 or PARP cleavage in H460, H157 and A549 cells (Figure 1A). Therefore, NSCLC cells lacking LKB1 are also sensitive to AICAR-induced apoptosis, and the kinase activity of LKB1 is required to suppress cell death under such conditions.

### Metabolomics screen reveals a depletion of uridine independent of LKB1 status after AICAR treatment

Because AICAR is a cellular metabolite, we next carried out a metabolomics screen for alterations of other metabolites in LKB1-null cells after AICAR treatment. H460-pBabe and MEF-pBabe cells were treated with 1 mM AICAR for 4 hrs in quadruplicate, and cell lysates were analyzed on the metabolon GC/MS and LC-MS/MS platform, which identified 381 known compounds in H460 cells and 307 named biochemicals in MEF cells. Statistically significant alterations observed in H460-pBabe (*p* < 0.05) and MEF-pBabe (*p* < 0.01) cells treated with or without AICAR are listed in Supplemental Table 1. Consistent with AICAR serving as a precursor in purine nucleotide synthesis, statistically significant increases in purine catabolite (xanthosine) were observed in both cell lines. However, this treatment did not result in significant alterations in GMP, AMP, ADP or ATP levels in H460-pBabe or LKB1-null MEF cells (Table 1). Hence, AICAR induced apoptosis in LKB1-null cells though a different mechanism than that of phenformin, which induces apoptosis through the depletion of intracellular ATP [16].

**Table 1:** Metabolomics screen of nucleotide pathway-related metabolites in isogenic H460 and MEF cells after AICAR treatment

Biochemical Name	H460-pBabe		MEF-pBabe		H460-LKB1		MEF-LKB1	
	Fold Change	p-value	Fold Change	p-value	Fold Change	p-value	Fold Change	p-value
**Purine metabolism**								
AICA ribonucleotide	**349.46**	**5.15E-12**	**319.21**	**0**	**490.61**	**2.57E-12**	**295.33**	**0**
xanthosine	**3.80**	**1.71E-07**	**2.66**	**3.62E-05**	**4.31**	**7.39E-08**	**6.94**	**4.82E-08**
guanosine 5′- monophosphate (5′-GMP)	4.02	0.20	1.48	0.32	1.53	0.65	0.87	0.87
adenosine 5′-monophosphate (AMP)	1.31	0.86	0.96	0.75	1.19	0.98	1.15	0.43
adenosine 5′-diphosphate (ADP)	1.46	0.35	0.67	0.07	0.81	0.30	1.13	0.62
adenosine 5′-triphosphate (ATP)	1.42	0.19	0.73	0.09	**0.61**	**0.0303**	0.76	0.63
**Pyrimidine metabolism**								
orotate	**115.06**	**0.01**			25.37	0.53		
uracil	**0.19**	**2.5E-06**	1.48	0.57	**0.38**	**0.0005**	0.55	0.11
uridine	**0.09**	**0.0003**	0.76	0.09	**0.24**	**0.01**	**0.69**	**0.03**
uridine monophosphate (5′ or 3′)	0.87	0.45	**0.61**	**0.01**	0.80	0.31	**0.53**	**0.0036**
uridine 5′-diphosphate (UDP)	0.72	0.12			**0.52**	**0.01**		
uridine 5′-triphosphate (UTP)	**0.44**	**0.0001**			**0.30**	**3.65E-06**		
cytidine	**0.15**	**9.05E-08**	0.86	0.47	0.77	0.13	**0.26**	**0.0012**
cytidine 5′-monophosphate (5′-CMP)	**0.76**	**0.04**	0.84	0.22	0.92	0.41	**0.67**	**0.02**
cytidine 5′-diphosphocholine	**0.48**	**0.01**	0.9	0.38	0.60	0.05	0.77	0.07

Fold Change = AICAR-treated sample/untreated-sample. P-value was calculated by two-way ANOVA contrast. Statistically significant alterations are bolded.

Uridine displayed the most statistically significant decrease in H460-pBabe cells after AICAR treatment, and the depletion of uridine-related metabolites, such as UTP, was also observed in MEF cells (Table 1). This is consistent with previous observations that the accumulation of intracellular ZMP leads to the depletion of pyrimidine nucleotide pools in mammalian cells [17]. This conclusion is also supported by a dramatic increase in the pyrimidine biosynthetic intermediate orotate in H460 cells and significant reductions in UTP, UDP, uridine, cytidine, and CMP levels as compared to controls (Table 1). This effect on pyrimidine metabolism is expected to have significant impact on DNA and RNA metabolism. The depletion of uridine and its related metabolites were also observed in H460 and MEF cells with wild type LKB1 expression (Table 1), indicating that AICAR-induced depletion of uridine was not prevented by LKB1. In addition, the observed increase in phosphoethanolamine in both H460 and MEFs following AICAR treatment may reflect an inhibition of phospholipid synthesis. This may be due to reduced levels of CTP as significant reductions in cytidine, CMP, and CDP-choline were observed in H460 cells treated with AICAR (Supplemental Table 1).

### Uridine is capable of rescuing AICAR-induced apoptosis in LKB1-null cells

**Figure 2:**
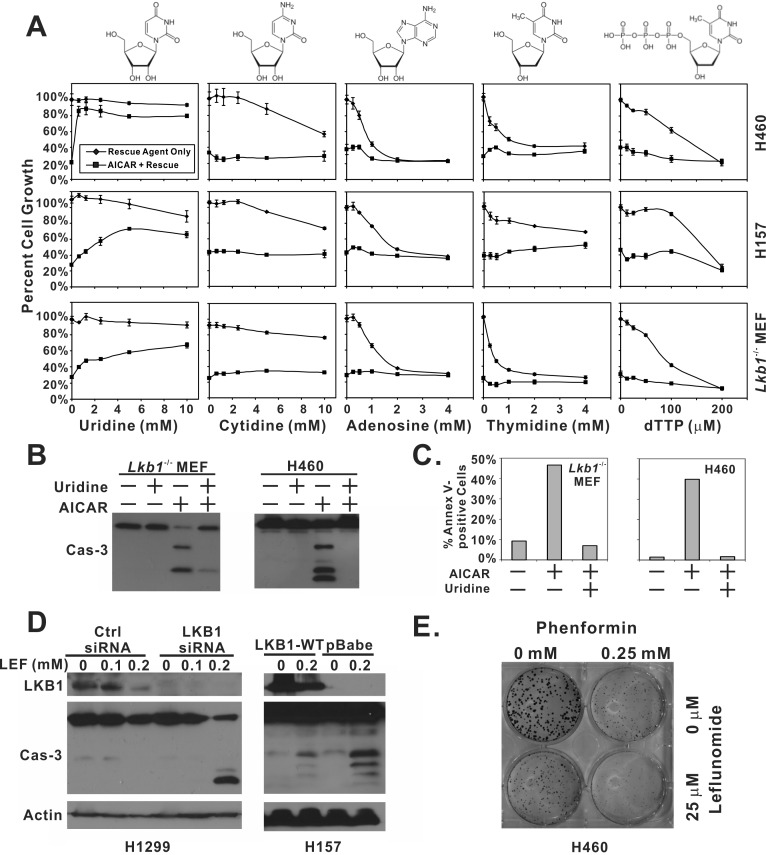
Depletion of uridine is responsible for AICAR-induced apoptosis in LKB1-null cells **A.** H460, H157 and *Lkb1*^−/−^ MEF cells were seeded in 96-well plates, and treated with the indicated concentration of uridine, cytidine, adenosine, thymidine, dTTP alone, or their respective combination with 1 mM AICAR. Plates were subjected to SRB assay 48 hrs after treatment. Reactions were carried out in quadruplicates, and the error bars represent one standard deviation. **B.** *Lkb1*^−/−^ MEF and H460 cells were seeded in 6-well plates and treated with 1 mM AICAR alone, 5 mM uridine alone or their combination. Lysates were collected 24 hrs after treatment for immunoblot analysis of total caspase-3. **C.** For Annexin-V and 7AAD analysis of apoptosis, *Lkb1*^−/−^ MEF and H460 cells were seeded in 6-well plates, and treated with 1 mM AICAR alone, 5 mM uridine alone, or their combination. Both floating and attached cells were collected 24 hrs after treatment and subjected to flow analysis. Percentage of Annexin-V positive cells was reported. **D.** Isogenic H157-pBabe and H157-LKB1-WT cells were treated with 0.2 mM of leflunomide for 48 hrs. Cell lysates were collected and analyzed by immunoblot with indicated antibodies. LKB1 was depleted in H1299 cells by siRNA and treated with 0, 0.1, or 0.2 mM of leflunomide for 48 hrs. Cell lysates were collected for immunblot of LKB1 and total caspase-3. **E.** H460 cells were treated with 0.25 mM phenformin, 25 μM leflunomide, or their combination for 10 days in a colony formation assay.

We next tested whether the inclusion of various nucleoside precursors was able to alleviate 1 mM AICAR-induced growth arrest and apoptosis in LKB1-null H460, H157, and MEF cells (Figure 2A). The inclusion of uridine in the culture media was sufficient to restore AICAR-inhibited growth from 21% to 80% in H460 cells and from 37% to 66% in H157 cells. This phenomenon appeared to be universal because a similar rescue effect was observed in LKB1-null MEF cells. The inclusion of uridine also prevented AICAR-induced PARP and caspase-3 cleavage in LKB1-null cells (Figure 2B). The ability of uridine to protect AICAR-treated cells from apoptosis was also supported by an Annexin-V/7AAD flow cytometry analysis (Figure 2C).

In contrast, other nucleoside precursors, cytidine, adenosine, and thymidine, failed to alleviate AICAR-mediated growth suppression. A previous synthetic lethality screening study revealed that “thymineless death” preferentially induces apoptosis in LKB1-null cells, and such death can be rescued by the inclusion of dTTP in culture media [18]. In our model, dTTP failed to reverse AICAR-induced growth suppression, indicating that thymineless death was not involved in AICAR-induced apoptosis in LKB1-null cells (Figure 2A).

We next tested whether inhibition of the *de novo* production of ribonucleotide uridine monophosphate (rUMP) preferentially induces apoptosis in LKB1-null cells. Leflunomide is an antirheumatic drug, and its active metabolite, A77-1726, inhibits human dihydroorotate dehydrogenase (hDHODH) that converts dihydroorotate to orotate, a precursor of rUMP. Caspase-3 cleavage after leflunomide treatment was preferentially observed in LKB1-depleted H1299 cells or LKB1-null H157-pBabe cells but not in their isogenic counterparts that express wild-type LKB1 (Figure 2D). These data are consistent with the notion that LKB1-deficient cells are sensitive to depletion of uridine-derived metabolites. Interestingly, we also noticed a consistent decrease in the amount of total LKB1 protein in both H1299 and H157-LKB1-WT cells after leflunomide treatment. The mechanism underlying this decrease was not known, but it was not sufficient to promote caspase-3 cleavage in H1299 cells.

Phenformin was recently shown to specifically induce cell death in LKB1-null cells [16], but uridine failed to rescue phenformin-induced growth suppression (Supplemental Figure 2). This data is consistent with our metabolite profile analysis which suggested that AICAR induces cell death through a different mechanism. This notion was further supported by colony formation assay, which indicated that the combination of phenformin and leflunomide was more effective in suppressing the growth of H460 cells than either agent alone (Figure 2E).

### AICAR treatment suppressed pre-rRNA synthesis in LKB1-null cells

**Figure 3:**
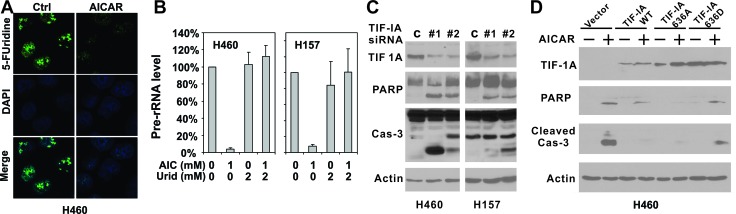
Disruption of TIF-IA mediated pre-rRNA synthesis is responsible for AICAR-induced apoptosis in LKB1-null/depleted cells **A.** H460 and H157 cells were plated on glass cover slips and treated with 1 mM AICAR for 12 hrs. Cells were labeled with 2 mM 5-fluorouridine before immunofluorescence analysis. **B.** H460 and H157 cells were seeded in 60 mm dishes and treated with 1 mM AICAR, 2 mM uridine alone, or their combination. RNA was extracted with Trizol 12 hrs after AICAR treatment. Pre-RNA level was assessed by real-time PCR amplifying pre-45S ribosomal RNA using 18S rRNA as control. **C.** H460 and H157 cells were seeded in 6-well plates and treated with TIF-IA siRNA or control siRNA. Cell lysates were collected 48 hrs after transfection for immunoblot analysis and total caspase-3 was checked. **D.** H460 cells were stably infected with retrovirus containing the indicated TIF-IA wild-type or mutant constructs. Cells were treated with 1 mM AICAR for 24 hrs. Cell lysates were analyzed by immunoblot using indicated antibodies, including the one against cleaved caspase-3.

Because uridine is the only nucleoside precursor capable of rescuing AICAR-induced apoptosis, AICAR treatment mostly likely induces apoptosis in LKB1-null cells through the disruption of RNA transcription rather than DNA replication. More than half of the total RNA synthesis inside a cell is used to produce ribosomal RNAs (rRNA) [4], which led us to investigate whether AICAR treatment disrupts pre-rRNA synthesis. The effect of AICAR treatment on pre-rRNA synthesis in LKB1-null H460 and H157 cells was assessed by immunofluorescence of 5-fluorouridine incorporation and quantitative real-time PCR (Figures. 3A and 3B). 5-fluorouridine is preferentially incorporated into rRNA and is commonly used as a marker for rRNA synthesis [19-21]. Our immunofluorescence analysis of 5-fluorouridine revealed extensive nucleolar staining in untreated cells (Figure 3A), which is consistent with the labeling of rRNA in the nucleolus. Treatment with 1 mM AICAR for 12 hrs resulted in a significant downregulation of 5-fluorouridine staining in the nucleoli, suggesting that AICAR suppressed pre-RNA synthesis. This was confirmed by quantitative real-time PCR analysis of pre-rRNA levels, which indicated that 1 mM AICAR treatment for 12 hours resulted in more than 90% decreases in pre-rRNA synthesis in both H460 and H157 cells (Figure 3B). qPCR analysis also indicated that the inclusion of 2 mM uridine was sufficient to restore pre-rRNA synthesis with AICAR treatment (Figure 3B).

### TIF-IA is an essential antagonist of AICAR-induced apoptosis in LKB1-null cells

The binding of TIF-IA/RNA Pol I to the UBF/SL1 complex is essential for the initiation of pre-rRNA synthesis, and genetic inactivation of TIF-IA promotes p53-mediated apoptosis [9]. We next directly suppressed pre-rRNA synthesis by depleting TIF-IA using siRNA in order to determine whether LKB1-null cells are prone to apoptosis after the disruption of pre-RNA synthesis. Two independent siRNAs were used to deplete TIF-IA protein expression in LKB1-null H460 and H157 cells. In both cell lines, TIF-IA depletion led to significant increases in PARP and caspase-3 cleavage in the absence of AICAR treatment (Figure 3C), indicating that the suppression of TIF-IA expression was sufficient to induce apoptosis in LKB1-null cells. In contrast, the stable overexpression of a wild-type TIF-IA in H460 cells significantly attenuated caspase-3 cleavage, and partially attenuated PARP cleavage (Figure 3D, lanes 3 & 4). Interestingly, such rescue was not observed when we overexpressed a TIF-IA 636D mutant (Figure 3D, lanes 7&8). The overexpression of a TIF-IA 636A mutant, in contrast, completely abolished PARP and caspase-3 cleavage (Figure 3D, lanes 5&6). In summary, these data suggest that TIF-IA plays a role in AICAR-induced apoptosis in LKB1-null cells.

### LKB1 promotes nuclear accumulation of TIF-IA to maintain pre-rRNA synthesis after AICAR treatment in H157 cells

**Figure 4:**
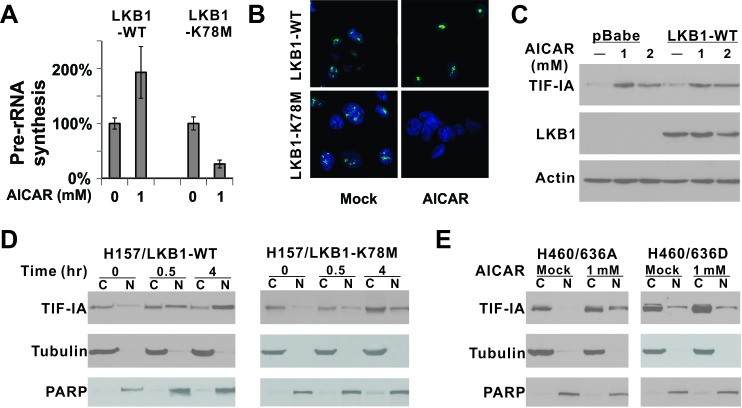
LKB1 kinase activity is required for the nuclear accumulation of TIF-IA after AICAR treatment **A.** Pre-rRNA synthesis was evaluated in H157-LKB1-WT or H157-LKB1-K78M cells by quantitative real-time PCR. K78M: Kinase-dead mutant. **B.** Pre-rRNA synthesis was evaluated by 5-fluorouridine incorporation. **C.** Alteration in endogenous TIF-IA protein level after 1 or 2 mM AICAR treatment for 24 hrs was evaluated by immunoblot in isogenic cells. **D.** Time course analysis of TIF-IA cellular localization after 1 mM AICAR treatment in H157 isogenic cells. **E.** Nuclear localization of ectopically-expressed 636A and 636D TIF-IA mutant in isogenic H460 cells after 1 mM AICAR treatment for 24 hrs was evaluated by immunoblot analysis. C: Cytoplasmic fraction; N: Nuclear fraction.

Results from our cell line panel indicated that AICAR-induced apoptosis was attenuated by ectopic overexpression of a wild-type LKB1 but not a kinase dead LKB1. We next evaluated pre-rRNA synthesis in isogenic cells of H157 background and found that pre-rRNA synthesis was not suppressed but elevated in cells expressing wild-type LKB1 (Figures 4A and 4B). Interestingly, TIF-IA protein level was elevated in both isogenic cells after 1 mM or 2 mM AICAR treatment (Figure 4C). However, the increased TIF-IA was found to preferentially accumulate in the nucleus in LKB1-WT expressing cells but not in kinase-dead mutant expressing cells (Supplemental Figure 3). A time-course analysis revealed that TIF-IA started to accumulate in the nucleus 30 mins after AICAR treatment in LKB1-WT cells, while such accumulation was significantly attenuated in LKB1-K78M cells (Figure 4D). Therefore, LKB1 activates pre-rRNA synthesis by actively promoting the nuclear accumulation of TIF-IA in H157-LKB1-WT cells after AICAR treatment.

We also evaluated the nuclear localization of ectopically-expressed TIF-IA mutants in H460 cells after AICAR treatment (Figure 4E). The TIF-IA 636A mutant was mostly localized in the cytosol, and AICAR treatment led to its nuclear accumulation. In contrast, the TIF-IA/635D mutant was detectable in the nuclear fraction under untreated conditions, but it failed to accumulate in the nucleus after AICAR treatment (Figure 4E). This failure is consistent with our observation that this mutant cannot rescue AICAR-induced apoptosis in H460 cells (Figure 3D).

It should be noted that TIF-IA did accumulate slowly in the nucleus of H157/LKB1-K78M cells after AICAR treatment (compare Figure 4D, right panel, lane 2 to lane 6). However, this increase in nuclear TIF-IA was not sufficient to restore pre-rRNA synthesis as demonstrated in Figure 4A and 4B. Therefore, TIF-IA from LKB1-null cells may also lack additional modification(s) to restore pre-rRNA synthesis even if it enters the nucleus.

### Overexpression of wild-type LKB1 rescues AMPK-null MEF cells from AICAR-induced apoptosis

**Figure 5:**
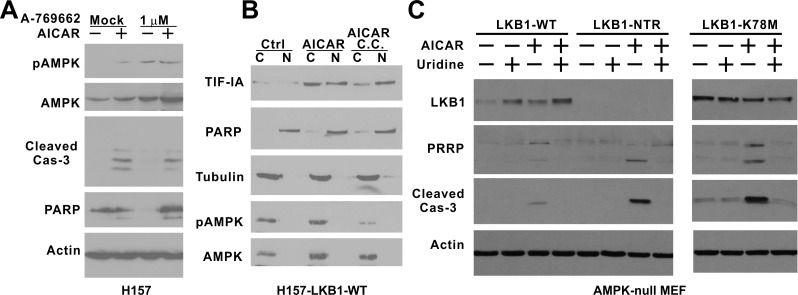
The kinase activity of LKB1 is required to rescue AMPK-null MEF cells from AICAR-induced apoptosis **A.** H157 cells were treated with 1 mM AICAR, 1 μM A-769662 or their combination for 24 hrs. **B.** H157-LKB1-WT cells were pre-treated with 10 μM compound C followed by 1 mM AICAR treatment for 4 hrs. Nuclear (N) and cytosolic (C) fractions were analyzed by immunoblot with indicated antibodies. **C.** AMPK-null MEF cells were infected with retrovirus containing the indicated LKB1 constructs to obtain stable cell lines. NTR: N-terminal Region; K78M: Kinase-dead mutant. Cells were treated 0.25 mM uridine, 0.25 mM AICAR or their combination for 24 hrs. Cell lysates were analyzed by immunoblot with indicated antibodies, including the one against cleaved caspase-3.

AMPK-null MEF cells were recently found to be also prone to AICAR-induced apoptosis [22]. Compared to LKB1-null MEF cells, AMPK-null MEFs are more sensitive to AICAR, and 0.25 mM AICAR treatment is sufficient to induce caspase-3 and PARP cleavage (Supplemental Figure 4A). In addition, we found that uridine was also sufficient to rescue AICAR-induced caspase-3 and PARP cleavage in this cell line (Supplemental Figure 4A). This led us to test the notion that the activation of AMPK in an LKB1-deficient background is sufficient to block AICAR-induced caspase-3 cleavage. A-769662 has been shown to activate AMPK in an LKB1-deficient background, and the treatment of H157 cells with 1 μM A-769662 significantly induced AMPK phosphorylation at Thr172 (Figure 5A). However, A-769662 failed to block AICAR-induced caspase-3 or PARP cleavage, indicating that the activation of AMPK in LKB1-null background was not sufficient to block AICAR-induced apoptosis. Compound C is commonly used for AMPK inhibition, and it significantly attenuated AMPK phosphorylation in H157-LKB1-WT cells after AICAR treatment (Figure 5B). This treatment, however, did not decrease the accumulation of TIF-IA in the nucleus after AICAR treatment, suggesting that the kinase activity of AMPK is not required for the nuclear accumulation of TIF-IA.

Because AMPK kinase activity was not required to protect cells against AICAR-induced apoptosis, we next used a genetic approach to evaluate the possibility that LKB1 is capable of protecting cells against AICAR-induced apoptosis in the absence of AMPK. We obtained AMPK-null MEF cells from Dr. Viollet [22], and overexpressed various LKB1 types in these cells. Interestingly, the overexpression of wild-type LKB1, but not LKB1-NTR truncated or LKB1-K78M mutants, significantly attenuated caspase-3 and PARP cleavage induced by 0.25 mM AICAR treatment (Figure 5C). Cell cycle analysis indicated that overexpression of LKB1 was sufficient to eliminate the SubG1 cell population after AICAR treatment (Supplemental Figure 4B). In addition, there was a significant decrease in the Annexin V positive cell population in AnnexinV/7AAD flow analysis in LKB1-WT expressing cells (Supplemental Figure 4C). It is important to note this protective effect was most pronounced at a low concentration (0.25 mM) of AICAR treatment. Nevertheless, this genetic evidence suggests that the over-expression of kinase-active LKB1 is capable of attenuating AICAR-induced apoptosis in AMPK-null MEF cells.

## DISCUSSION

**Figure 6:**
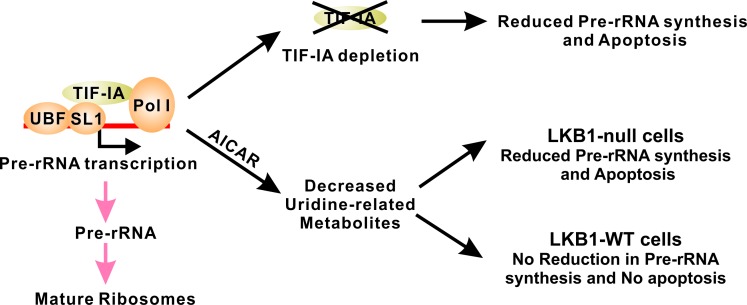
LKB1 promotes cell survival in uridine-depleted condition by maintaining pre-RNA synthesis TIF-IA-mediated pre-RNA synthesis is tightly regulated and genetic inactivation of TIF-IA is sufficient to promote apoptosis. AICAR treatment leads to the decrease of uridine-related metabolites. In LKB1-null cells, such decrease also results in the downregulation of pre-rRNA synthesis and cell death. Wild-type LKB1 is capable of maintaining pre-rRNA synthesis under these conditions to maintain cell survival.

TIF-IA is essential for the initiation of pre-rRNA transcription through its interactions with RNA Pol I and UBF/SL1 at the ribosomal DNA promoter, and it is hyperactivated by various oncogenic signaling pathways, such as ATK, ERK/RSK and mTOR, in human cancer [6-8]. Furthermore, genetic inactivation of TIF-IA has been shown to promote p53-dependent apoptosis [9]. We have shown here that AICAR treatment leads to a depletion of uridine and other uridine-related metabolites through metabolic profile analysis, which results in a significant downregulation of pre-rRNA synthesis in LKB1-deficient cells (Figure 6). We also demonstrated that this downregulated pre-rRNA synthesis is responsible for AICAR-induced apoptosis because the overexpression of a wild-type or 636A TIF-IA is sufficient to attenuate this apoptosis in LKB1-null H460 cells. It is important to note that AICAR-induced apoptosis is not p53-dependent in NSCLC as it was a universal phenomenon in our panel of cell lines, many of which contain p53 inactivation mutations (H1299, H1792 and H157). The kinase activity of LKB1 is the only biological means to protect NSCLC cells against AICAR treatment, and we demonstrated that this activity is capable of promoting TIF-IA-mediated pre-rRNA synthesis following AICAR treatment in H157-LKB1-WT cells by actively recruiting TIF-IA into the nucleus. In addition, we demonstrated that a phosphor-mimetic 636D TIF-IA mutant is not accumulated in the nucleus after AICAR treatment in the LKB1-null background and cannot rescue AICAR-induced apoptosis (Figure 4D and 3D), suggesting that the phosphorylation of TIF-IA at its C-terminus may regulate its cellular localization. The next logical step of this line of study is to determine which protein complex is involved in the regulation of TIF-IA cellular localization, and whether the interaction between this complex and TIF-IA is regulated by the kinase activity of LKB1. In the absence of LKB1, TIF-IA does slowly accumulate in the nucleus but such nuclear TIF-IA is not sufficient to restore pre-rRNA synthesis (Figures 4A, 4B and 4D). Therefore, LKB1 also appears to regulate other aspects of TIF-IA-mediated pre-RNA synthesis in addition to its cellular localization. LKB1 is best known as a tumor suppressor but our study has revealed a novel pro-survival function of LKB1 under uridine downregulated conditions (Figure 6).

A surprising finding of this study is the role of AMPK in this process. AMPK is the best known downstream substrate of LKB1, and AMPK-null MEF cells were extremely sensitive to AICAR treatment [22]. Thus, a simple assumption is that AMPK acts downstream to regulate TIF-IA-mediated pre-rRNA synthesis [23]. However, AMPK has been shown to directly phosphorylate TIF-IA at serine 635, and this phosphorylation disrupts binding of TIF-IA/RNA Pol I to the UBF/SL1 complex to suppress pre-rRNA synthesis [21]. Furthermore, chemical inhibition of AMPK kinase activity did not prevent LKB1-mediated nuclear accumulation of TIF-IA in H157 cells (Figure 5B). These findings suggest that AMPK does not act downstream of LKB1 to protect cells from AICAR-induced apoptosis. This notion was supported by our genetic analysis which indicated that only the overexpression of wild-type LKB1 attenuates AICAR-induced apoptosis in AMPK-null MEF cells (Figure 5C), demonstrating that this LKB1 kinase-dependent prosurvival function is distinct from the previously known prosurvival function of the LKB1/AMPK metabolic checkpoint.

Our findings also have an important clinical implication. The nucleolus is an emerging therapeutic target for cancer treatment [2, 24-26]. At least three types of FDA-approved anti-cancer agent were found to also suppress rRNA synthesis: (i) Temsirolimnus/everolimus (mTOR inhibitors) inhibit RNA Pol I transcription [27], (ii) topotecan/irinotecan modulate early rRNA processing [28], and (iii) 5-fluorouracil (5-FU) impairs late rRNA processing [29]. Of the reagents that are in phase I or phase II clinical development, CX-3543 disrupts Pol I transcription elongation [30], CX-5461 inhibits Pol I initiation [31], and ellipticine impairs SL-1 rDNA promoter binding [32]. Yet, the concept of targeting rRNA synthesis for cancer therapy has a major conceptual obstacle, i.e. rRNA synthesis is an essential housekeeping process in normal cells, and all reagents described above cannot discriminate between highly proliferating normal cells and cancer cells. Our findings suggest that cancer cells lacking LKB1 have lost the ability to maintain pre-rRNA synthesis under uridine down-regulated conditions, and thus are sensitive to agents that deplete the intracellular uridine pool, such as leflunomide, which inhibits the *de novo* synthesis of UMP. It will be of significant interest in the future to determine whether such reagents can be used to specifically suppress rRNA synthesis in LKB1-null cancer cells. Recently, phenformin has emerged as a promising agent to eliminate LKB1-null cancers through the depletion of intracellular ATP [16]. Our data indicate that the combination of leflunomide and phenformin is more effective in suppressing colony formation than either agent alone, suggesting that various LKB1-deficiency-based tumor vulnerabilities may be targeted simultaneously in the clinic.

In summary, we have demonstrated that AICAR treatment leads to the depletion of intracellular uridine and the production of its derivative, such as UTP, which ultimately results in the downregulation of pre-rRNA synthesis in LKB1-null NSCLC cells and promotes apoptosis. In LKB1-wild type cells, the kinase activity of LKB1 is capable of promoting TIF-IA mediated pre-rRNA synthesis under such conditions to promote cell survival. Our study has revealed that LKB1-null NSCLC cells have a defect in the regulation of pre-rRNA synthesis which may be exploited therapeutically to enable their elimination.

## MATERIALS AND METHODS

### Reagents and antibodies

5-amino-1-β-D-ribofuranosyl-imidazole-4-carboxamide (AICAR) was purchased from Toronto Research Chemicals Inc (North York, Canada). Uridine, cytidine, adenosine, thymidine, deoxythymidine triphosphate (dTTP), uracil, phenformin and 5-fluorouridine were purchased from Sigma-Aldrich Co. LLC, (St Louis, MO). Mouse monoclonal antibody against LKB1 was purchased from Abcam (Cambridge, MA). Antibodies against AMPKα, phospho-AMPKα (Thr172), total caspase 3, cleaved caspase-3, PARP, and phospho-acetyl-coA carboxylase (phospho-ACC, Ser79) were purchased from Cell Signaling Technology (Beverly, MA). Mouse monoclonal antibody and polyclonal antibody against TIF-IA were purchased from Santa Cruz Biotechnology Inc (Dallas, TX) and Abcam, respectively. Mouse polyclonal anti-beta-actin antibody was purchased from Sigma.

### Cell culture and retrovirus infection

NSCLC cell lines H1299, H460, H157 and A549 were purchased from the American Type Culture Collection (ATCC). Cells were maintained according to the instructions from ATCC. The identities of these cell lines were verified by genotyping service at Emory University. LKB1-null and AMPK-null MEF cells were gifts from Profs. DePinho and Viollet, respectively. The LKB1-WT and LKB1-K78M pBabe vectors were purchased from Addgene (Cambridge, MA). GFP-tagged LKB1 and the various domains and mutations were subcloned from a pEGFP-C1 vector into pBabe-puro. H460, H157, A549 and AMPK-null MEF cells were infected with pBabe-based retroviruses encoding LKB1-WT, LKB1-K78M or vector control. LKB1-null MEF cells were infected with GFP-tagged LKB1 and various domains and mutations.

TIF-IA plasmid was obtained from Addgene (plasmid#17661), and TIF-IA 636A and 636D were generated by site-directed mutagenesis using primers described in Supplemental Table 2. Wild type and mutant TIF-IA constructs were generated by polymerase chain reaction and subcloned into pLHCX retroviral vector. To generate TIF-IA rescue H460 cell lines, TIF-IA wild type, 636A, and 636D were cloned into the retroviral vector pLHCX (Clontech, Mountain View, CA). The constructs were cotransfected with pAmpho cassette in GP2-293 cells. Retrovirus was harvested 48 hrs after transfection. H460 cells were infected with harvested retrovirus and were selected by Hygromycin B (300 μg/ml) (Thermo Fisher Scientific Inc., Waltham, MA) for 2 weeks.

### Metabolomic screen

Isogenic H460-pBabe/H460-LKB1 and MEF-pBabe/MEF-LKB1 cells were treated with 0 or 1 mM AICAR for 4 hrs. Reactions were carried out with 4 replicates for each treatment condition. Cell lysates were extracted and prepared for analysis using Metabolon's standard solvent extraction method. The extracted samples were split into equal parts for analysis on the GC/MS and LC-MS/MS platforms. Fold changes were calculated as AICAR-treated value divided by untreated value. P-values were calculated by two-way ANOVA contrasts. Sample processing and statistical analysis were carried out by Metabolon Inc (Durham, NC).

### Immunoblot analysis

Preparation of whole cell protein lysate followed by immunoblot were performed as described previously [33]. Briefly, total protein lysate (40 μg) was electrophoresed through 12% denaturing polyacryamide gels and then transferred to PVDF membrane (Bio-Rad, Hercules, CA) with a wet-transfer technique according to the manufacturer's protocol. The membranes were blocked with 10% non-fat milk, and then probed with antibodies against the specified proteins. The same blots were used for probing with antibodies against phospho-proteins and the corresponding total protein. Actin was used as a loading control.

### Small interfering RNA (siRNA) treatment

LKB1 and TIF-IA siRNA duplexes were purchased from Sigma. The sequences of these siRNAs are provided in Supplemental Table 2. Gene silencing was achieved as described previously [34]. Briefly, cells were grown to 50∼60% confluence in 6-well plates, Lipofectamine reagent (Invitrogen, Grand Island, NY) was incubated with Opti-MEM reduced serum medium (Invitrogen) for 10 mins, and a mixture of siRNA was then added. After incubation for 20 mins at room temperature, the mixture (300 μl) followed by 700 μl Opti-MEM was added to each well. After incubating for 6 hrs, the media was replaced with complete medium. 80 pmol of siRNA was used per well. Cells were treated with the desired chemical for the desired time, and then harvested for immunoblot, cell cycle or apoptosis analysis.

### Cell cycle analysis

Cells were seeded in 6-well plates, subjected to the desired treatment, and then trypsinized and collected in 15 ml tubes. Cells were washed twice with PBS, then fixed with 70% ethanol for 30 mins. After removal of ethanol, the cells were stained with PI/RNASE staining kit (BD Biosciences, San Jose, CA) for 30 mins at room temperature in the dark, and then analyzed by FACS analysis (BD Biosciences). A total of 10,000 gated cells were acquired for each analysis. Results were analyzed by FlowJo version 7 software (FlowJo. LLC, Ashland, OR).

### Apoptosis analysis

Apoptosis was measured by flow cytometry with the Annexin V-PE Apoptosis Detection kit (BD Biosciences). Cells were seeded in 6-well plates and subjected to the desired treatment. Both floating and attached cells were collected, washed twice with cold PBS and resuspended in 1x binding buffer. 100 μl of the cell suspension (representing 5 × 10^5^ cells) was transferred to a culture tube followed by adding 5 μl of Annexin V-PE and 5 μl of 7-AAD, mixed by vortex, and then incubated for 15 mins at room temperature in the dark. Apoptosis analysis was carried out by FACS (BD Biosciences). 10,000 cells were acquired for each analysis. Results were analyzed by FlowJo version 7 software (FlowJo. LLC).

### SRB assay

2000-3000 cells were seeded in 96-well plates, treated with the desired chemical for 48 hrs and subjected to SRB assay as described previously [35].

### Quantitative real-time RT-PCR analysis

Cellular RNA was isolated with Trizol (Invitrogen). Total RNA was reverse transcribed to cDNA, and qPCR was carried out in a 20 μl volume using iQ SYBR green supermix (Bio-Rad) on a Bio-Rad iCycler (Bio-Rad). qPCR primers are included in Supplemental Table 2.

### Nuclear and cytosolic fraction analysis

Nuclear and cytosolic fractions were extracted from cells using NE-PER™ Nuclear and Cytoplasmic Extraction Reagents (Life Technologies, Grand Island, NY) with modifications as follows: after transferring the supernatant (cytoplasmic extract), the pellet was washed twice more with pre-cold PBS and the supernatant discarded after centrifuging. An equal volume of each fraction was subjected to SDS-polyacrylamide gel electrophoresis (PAGE) analysis and Western blot. PARP and tubulin were used as a loading control.

### Immunofluorescence

10,000 cells were plated onto a 12 mm glass cover slip in a 24-well plate and incubated for 2 nights. The cells were treated with 1 mM AICAR for 12 hrs and then labeled with 2 mM 5-fluorouridine (Sigma) for 15 mins in the incubator. The coverslips were washed with PBS and fixed in 2% formaldehyde (Thermo Fisher Scientific Inc.) for 20 mins in the dark at room temperature. The fixed cells were permeabilized with methanol (Thermo Fisher Scientific Inc.) at −20°C for 10 mins. Coverslips were then blocked with 2% goat serum (Sigma) for 30 mins and incubated with the anti-BrdU antibody (Sigma) at 4°C overnight. The coverslips were washed with PBS and incubated with a secondary goat anti-mouse IgG/IgM (H+L) AlexaFluor^®^ 488 conjugated antibody (Life Technologies) for 1 hr at room temperature. After washing with PBS 3 times, cells were mounted using the Prolong Diamond anti-fade reagent with DAPI (Life Technologies). Images were obtained as single optical slides using a LSM510-Meta confocal microscope (Zeiss, Jena, Germany) equipped with a 63×1.3 oil immersion objective and excitation wavelengths of 405 nm and 488 nm.

## SUPPLEMENTARY MATERIAL TABLES AND FIGURES



## Abbreviations

The abbreviations used are:

ACC: acetyl-coA carboxylase
AICAR: 5-aminoimidazole-4-carboxamide riboside
AMPK: AMP-activate protein kinase
CK2α: casein kinase IIα
ERK: extracellular-signal-regulated kinase
hDHODH: human dihydroorotate dehydrogenase
LKB1: liver kinase B1
MEF: mouse embryonic fibroblast
mTOR: mammalian target of rapamycin
NSCLC: non-small cell lung cancer
PARP: poly ADP ribose polymerase
RNA Pol I: RNA polymerase I
rRNA: ribosomal RNA
RSK: ribosomal s6 kinase
rUMP: ribonucleotide uridine monophosphate
TIF-IA: RNA polymerase I-specific transcription initiation factor
ZMP: AICAr monophosphate.
