# Correction: LKB1 promotes cell survival by modulating TIF-IA-mediated pre-ribosomal RNA synthesis under uridine downregulated conditions

**DOI:** 10.18632/oncotarget.28843

**Published:** 2026-03-31

**Authors:** Fakeng Liu, Rui Jin, Xiuju Liu, Henry Huang, Scott C. Wilkinson, Diansheng Zhong, Fadlo R. Khuri, Haian Fu, Adam Marcus, Yulong He, Wei Zhou

**Affiliations:** ^1^Department of Hematology and Medical Oncology, Emory University School of Medicine, Atlanta, GA, USA; ^2^Department of Gastrointestinal Surgery, The First Affiliated Hospital of Sun Yat-sen University, Guangzhou, P.R. China; ^3^Graduate Program in Cancer Biology, Emory University, Atlanta, GA, USA; ^4^Department of Medical Oncology, Tianjin Medical University General Hospital, Tianjin, P.R.China; ^5^Department of Pharmacology, Emory University School of Medicine, Atlanta, GA, USA; ^6^Department of Pathology and Laboratory Medicine and Department of Human Genetics Emory University School of Medicine, Atlanta, GA, USA; ^*^These authors have contributed equally to this work

**This article has been corrected:** Due to an error in the authors’ inventory system, the pBabe-LKB1-mutant plasmid purchase from Addgene was mislabeled as K78M instead of K78I. Therefore, all the cell lines containing mutant LKB1 should be labeled K78I, not K78M, throughout this article. The authors declare that these corrections do not change the results or conclusions of this paper.

Original article: Oncotarget. 2016; 7:2519–2531. 2519-2531. https://doi.org/10.18632/oncotarget.6224

