# Supplementary material for: LKB1 promotes cell survival by modulating TIF-IA-mediated pre-ribosomal RNA synthesis under uridine downregulated conditions
Source: Oncotarget. 2015 Oct 25;7(3):2519–31. doi: 10.18632/oncotarget.6224 (PMC4823052; doi:10.18632/oncotarget.6224)
Supplement: Supplementary file 1 [file oncotarget-07-2519-s001.pdf]

LKB1 promotes cell survival by modulating TIF-IA-mediated pre-ribosomal RNA synthesis under uridine downregulated conditions

Supplementary Material

Supplemental Table 1. Metabolomics screen of isogenic H460 and MEF cells after AICAR treatment

	**H460-pBabe**		**MEF-pBabe**		**H460-LKB1**		**MEF-LKB1**	
Biochemical Name	Fold Change	p-value	Fold Change	p-value	Fold Change	p-value	Fold Change	p-value
**Statistically Significant Increases**								
AICA ribonucleotide	349.46	5.15E-12	319.21	0	490.61	2.572E-12	295.33	0
orotate	115.06	0.01			25.37	0.53		
phosphoethanolamine	6.85	5.8101E-05	1.50	0.03	6.47	9.2021E-05	8.58	3.0145E-08
xanthosine	3.80	1.7073E-07	2.66	3.6205E-05	4.31	7.3851E-08	6.94	4.8215E-08
5-methyltetrahydrofolate (5MeTHF)	1.87	8.4404E-05	0.85	0.02	1.14	0.25	0.92	0.19
S-adenosylhomocysteine (SAH)	1.33	0.0019	1.33	0.07	1.25	0.01	1.19	0.24
**Statistical Significant Decreases**								
pantothenate	0.85	0.01	0.79	0.01	0.73	8.3736E-05	1.11	0.23
uridine 5'-diphosphate (UDP)	0.72	0.12			0.52	0.01		
UDP-glucose	0.58	7.6817E-05	0.67	0.02	0.59	0.00008392	0.41	3.0675E-05
uridine 5'-triphosphate (UTP)	0.44	0.0001			0.3	3.6506E-06		
UDP-galactose	0.23	0.00002759	0.55	0.0014	0.22	1.4873E-05	0.34	5.4146E-06
glutamate, gamma-methyl ester	0.22	1.6212E-08	0.49	0.0007	0.15	1.6275E-09	0.64	0.01
glycerol 2-phosphate	0.21	0.0037	0.36	2.6346E-05	0.40	0.02	0.32	6.8112E-06
uridine	0.09	0.0003	0.76	0.09	0.24	0.01	0.69	0.03

Fold Change = AICAR-treated sample/untreated-sample. P-value was calculated by two-way ANOVA contrast.

Supplemental Table 2			
**siRNA**			
LKB1#1	5'-	GGACUGACGUGUAGAACAATT	-3'
LKB1#2	5'-	GCUCUUACGGCAAGGUGAA	-3'
TIF-1A#1	5'	AAUGCGUGUCAAAGGAGCUUGGUGU[dG][dA]	-3'
TIF-1A#2	5'	CGACACCGUGGUUUCUCAUGCCAAU[dT][dT]	-3'
**qPCR Primer**			
HuPre-rRNA-F	5'-	TGTCAGGCGTTCTCGTCTC	-3'
HuPre-rRNA-R	5'-	AGCACGACGTCACCACATC	-3'
18S-F	5'-	GAGGGAGCCTGAGAAACGG	-3'
18S-R	5'-	GTCGGGAGTGGGTAATTTGC	-3'
**site-directed mutagenesis primer**			
TIF-1A S636A-F	5'-	CCGAAGTCCTTCAGCTAGTGTGGGCTCCCC	-3'
TIF-1A S636A-R	5'-	GGGGAGCCCACACTAGCTGAAGGACTTCGG	-3'
TIF-1A S636D-F	5'-	CCGAAGTCCTTCAGATAGTGTGGGCTCCCC	-3'
TIF-1A S636D-R	5'-	GGGGAGCCCACACTATCTGAAGGACTTCGG	-3'

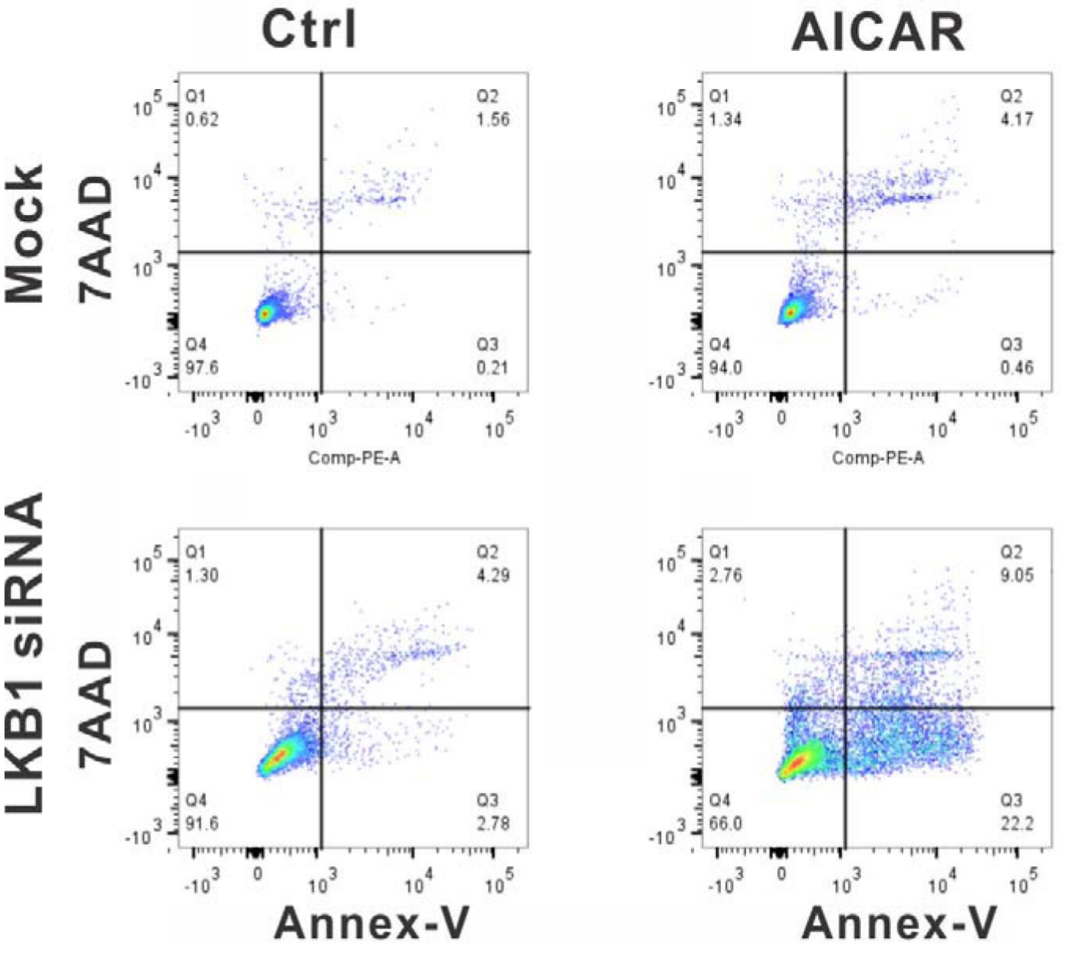

Supplemental Figure 1. LKB1 siRNA was used to deplete LKB1 in H1299 cells. Cells were treated with 2 mM AICAR for 48 hrs and analyzed for Annex-V/7AAD by flow cytometry.

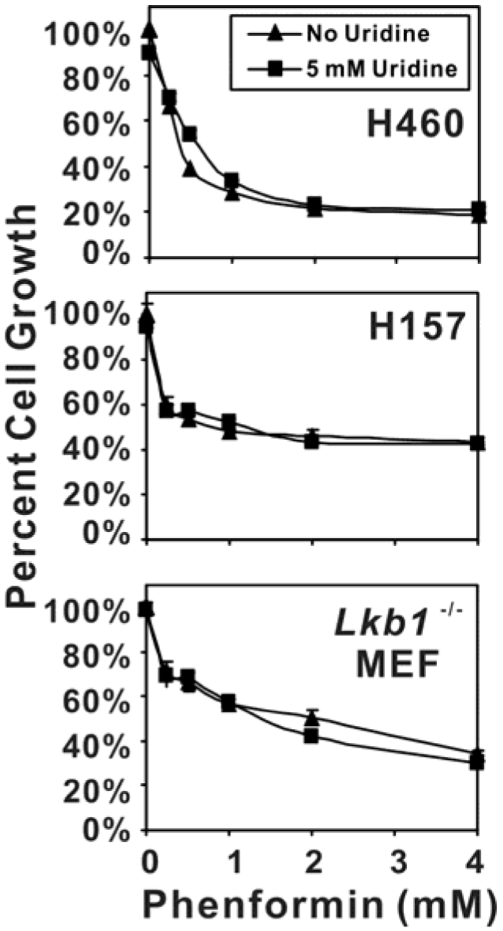

Supplemental Figure 2. Uridine fails to rescue phenformin induced growth inhibition in LKB1-null cells. H460, H157 and MEF *Lkb1*$^{-/-}$ cells were seeded in 96-well plates, and treated with the indicated concentration of phenformin, or its combination with 5 mM uridine. Plates were subjected to MTS assay 48 hrs after treatment. Reactions were carried out with 4 replicates for each treatment.

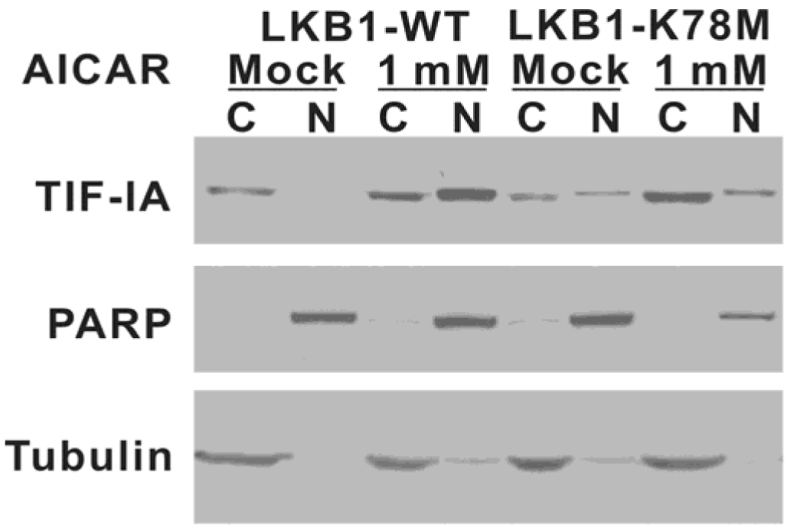

Supplemental Figure 3. Nuclear localization of TIF-IA in isogenic H157 cells after 1 mM AICAR treatment for 48 hrs was evaluated by immunoblot analysis. C: Cytoplasmic fraction; N: Nuclear fraction.

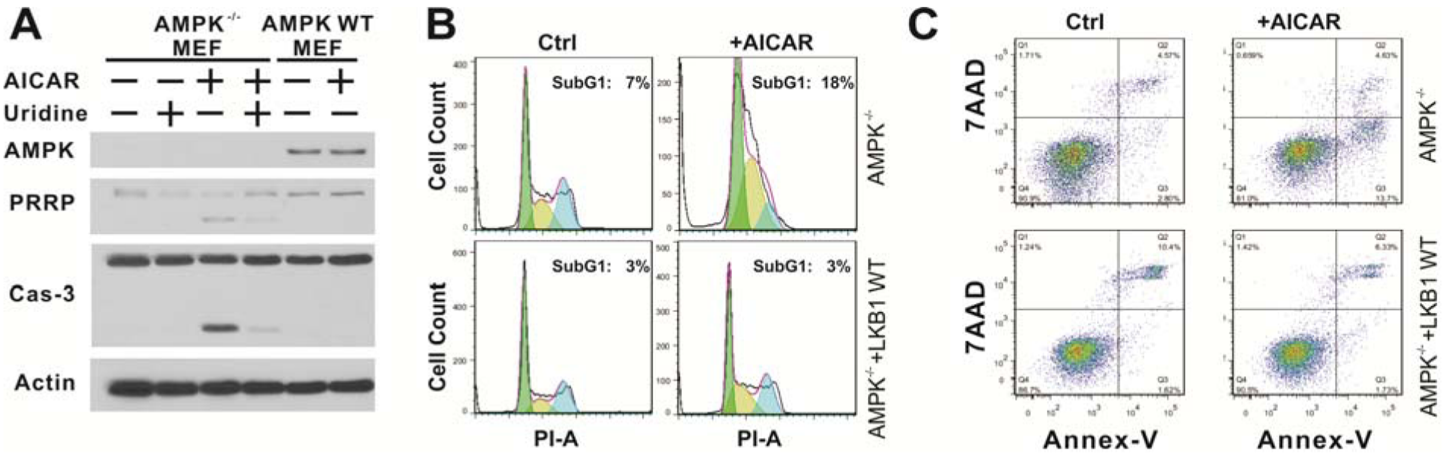

Supplemental Figure 4. AICAR induces apoptosis in AMPK-null MEF cells can be rescued by uridine or wild-type LKB1 expression. **A.** AMPK-null or wildtype MEF cells were seeded in 6-well plates and treated with 0.25 mM AICAR alone, 0.25 mM uridine alone or their combination. Lysates were collected 24 hrs after treatment for immunoblot analysis of indicated antibodies including total caspase-3. **B.** For cell cycle analysis, AMPK-null MEF and AMPK-null MEF cells with wild-type LKB1 expression were seeded in 6-well plates, and treated with 0.25 mM AICAR for 24 hrs. **C.** For Annexin-V and 7AAD analysis of apoptosis, both floating and attached cells were collected 24 hrs after 0.25 mM AICAR treatment and subjected to flow analysis.
